# Adjuvant music therapy for patients with hypertension: a meta-analysis and systematic review

**DOI:** 10.1186/s12906-023-03929-6

**Published:** 2023-04-06

**Authors:** Min Cao, Zhiyuan Zhang

**Affiliations:** 1 Shaoxing University Yuanpei College, No. 2799 Qunxian Zhong Road, Yuecheng District, Shaoxing, Zhejiang China; 2 Hunan international econonmics university, Hunan, China

**Keywords:** Music therapy, Hypertension, Blood pressure, Care, Treatment

## Abstract

**Background:**

High blood pressure, anxiety, depression and sleep disorder is very common in patients with hypertension. We aimed to perform a meta-analysis to evaluate the effects of adjuvant music therapy for patients with hypertension, to provide insights to the clinical management of hypertension.

**Methods:**

Two authors searched PubMed, Embase, Web of Science, the Cochrane Library, Chinese National Knowledge Infrastructure, China Biomedical Literature Database, Wanfang Databases for randomized controlled trials (RCTs) on the role of music therapy in hypertension up to Oct 15, 2022. RevMan 5.3 software was used for meta-analysis.

**Results:**

A total of 20 RCTs including 2306 patients were finally included. 1154 patients received music therapy. Meta-analysis showed that music therapy can effectively reduce the systolic blood pressure(MD = − 9.00, 95%CI: − 11.99~- 6.00), diastolic blood pressure(MD = -6.53, 95%CI: -9.12~- 3.93), heart rate (MD = -3.76, 95%CI: -7.32~- 0.20), self-rating anxiety scale (SAS) score(MD =-8.55, 95%CI: -12.04~-4.12), self-rating depression scale (SDS) score(MD = -9.17, 95%CI: -13.85~-5.18), Hamilton anxiety scale (HAMA), score(MD = -3.37, 95%CI: − 5.38~- 1.36), PSQI score(MD =-1.61, 95%CI:-2.30~- 0.93) compared with routine therapy in patients with hypertension(all P < 0.05). No publication bias in the synthesized outcomes were found (all P > 0.05).

**Conclusion:**

Music therapy can effectively control blood pressure and heart rate, reduce anxiety and depression levels, and improve sleep quality in hypertensive patients. Limited by the quantity and quality of included studies, the above conclusions need to be verified by more high-quality studies.

**Supplementary Information:**

The online version contains supplementary material available at 10.1186/s12906-023-03929-6.

## Background

Hypertension is the first risk factor for cardiovascular associated diseases. With the change of lifestyle and rhythm, the prevalence of hypertension in countries around the world is increasing year by year [1]. Negative emotions such as high-sodium diet, obesity, excessive drinking, and anxiety and depression are all risk factors for hypertension [2, 3]. Negative emotions such as anxiety and depression interact with hypertension. Previous epidemiological studies [4, 5] have found that about 38.5% of Chinese hypertensive patients are associated with anxiety, and 19.8% were associated with depression. Negative emotions such as anxiety and depression can affect the degree of hypertension and reduce the quality of life of patients by affecting the release of vascular endothelial factor, reducing vascular activity, and increasing vascular resistance [6–8]. Therefore, it is very important to focus on improving patients’ anxiety and depression while treating hypertension.

At present, there are many drugs for the treatment of hypertension in clinic. Although they can control hypertension to a certain extent, long-term use of drug use is easy to produce drug resistance, and it is easy to relapse after drug withdrawal. Nonpharmacological treatments can be effective in lowering blood pressure without other health risks. As one of the non-drug treatment methods, music therapy has been used as an adjuvant therapy in hypertension treatment. Currently, many scholars [9–11] have evaluated the psychological and physiological effects of music therapy on hypertensive patients, but the sample size of each study is small, and the research results remain different. Even though previous meta-analyses [12, 13] have analyzed the of the effect of music therapy on blood pressure in patients with hypertension, with more related studies published, updated meta-analyses on the role of music therapy for hypertention are needed. Therefore, this meta-analysis aimed to use the meta-analysis method to comprehensively evaluate the application effect of music therapy on hypertensive patients, in order to provide evidence-based basis for the treatment and management of hypertension.

## Methods

We conducted and reported this meta-analysis according to the preferred reporting items for systematic reviews and meta-analyses (PRISMA) statement [14].

### Document retrieval

Two authors searched PubMed, Embase, Web of Science, the Cochrane Library, Chinese National Knowledge Infrastructure (CNKI), China Biomedical Literature Database, Wanfang Databases for randomized controlled trials (RCTs) on the role of music therapy in hypertension. The retrieval time is from the establishment of the database to Oct 15, 2022. We used a combination of subject headings and free words to search. The language of searched and included publications were limited to English and Chinese. The search terms were as following: (“music” OR “music therapy” OR “sound therapy”) AND (“hypertension” OR “high blood pressure” OR “cardiovascular”)(Supplementary 1). In addition, we conducted a review search of relevant references in the included RCTs and important reviews to broaden the scope of the search.

### Inclusion and exclusion criteria

The inclusion criteria for this meta-analysis were: (1) study type: RCT design; (2) study population was the hypertensive patients, who met the diagnostic criteria for hypertension: Systolic blood pressure ≥ 140mmHg and / or diastolic blood pressure ≥ 90mmHg[15, 16]; (3) intervention measures: in addition to receiving routine treatment, patients in the music group accepted the music intervention. The patients in the control group only received the same routine treatment as the music therapy intervention group; (4) The study reported the corresponding outcome indicators, and the data could be extracted.

The exclusion criteria for this meta-analysis were: (1) studies with unclear diagnosis of hypertension or hypertension complicated with risk factors for other diseases; (2) research population were special populations such as pregnant women and military personnel; (3) Low-quality reports(significant data errors, study design problems and incomplete data); (4) reviews, cases and those literature reports with data that could not be extracted for analysis.

### Literature screening and data extraction

Literature screening and data extraction were conducted independently by two researchers in a blinded manner, and inconsistent literatures were reviewed and discussed. The following data were extracted according to the designed table: author, publication year, country, sample size, details of intervention measures and reported outcome indicators. The primary outcomes included systolic blood pressure, diastolic blood pressure. The secondary outcomes included heart rate, self-rating anxiety scale (SAS), self-rating depression scale (SDS), Hamilton anxiety scale (HAMA), Pittsburgh sleep quality index (PSQI).

### Quality assessment

The quality of the included literature was evaluated according to the risk of bias tool [17] recommended by Cochrane library. The tool includes following items: sequence generation, allocation concealment, blinding of participants and personnel, blinding of outcome assessment, incomplete outcome data, selective outcome reporting, and “other” issues. Every item can be rated as “low risk of bias”, “high risk of bias” or “unclear risk of bias” accordingly.

### Statistical analysis

We used Review Manager (RevMan) Version 5.3. software for meta-analysis. Measurement data were expressed as mean difference (MD) and 95% confidence interval (CI). In addition, we performed the heterogeneity test by the Q test, and combined with I^2^ to quantitatively judge the heterogeneity. If there was homogeneity among studies(I^2^ < 50%), a fixed-effects model was used to calculate the combined statistics; if there was heterogeneity(I^2^ ≥ 50%), random-effects model was used. Sensitivity analysis was performed by excluding articles one by one and then recombining the calculation. A funnel plot and Egger’s test were used to assess the publication bias of the pooled results. In this meta-analysis, P < 0.05 was considered to be statistically significant between groups.

## Results

### Study inclusion

**Fig. 1:**
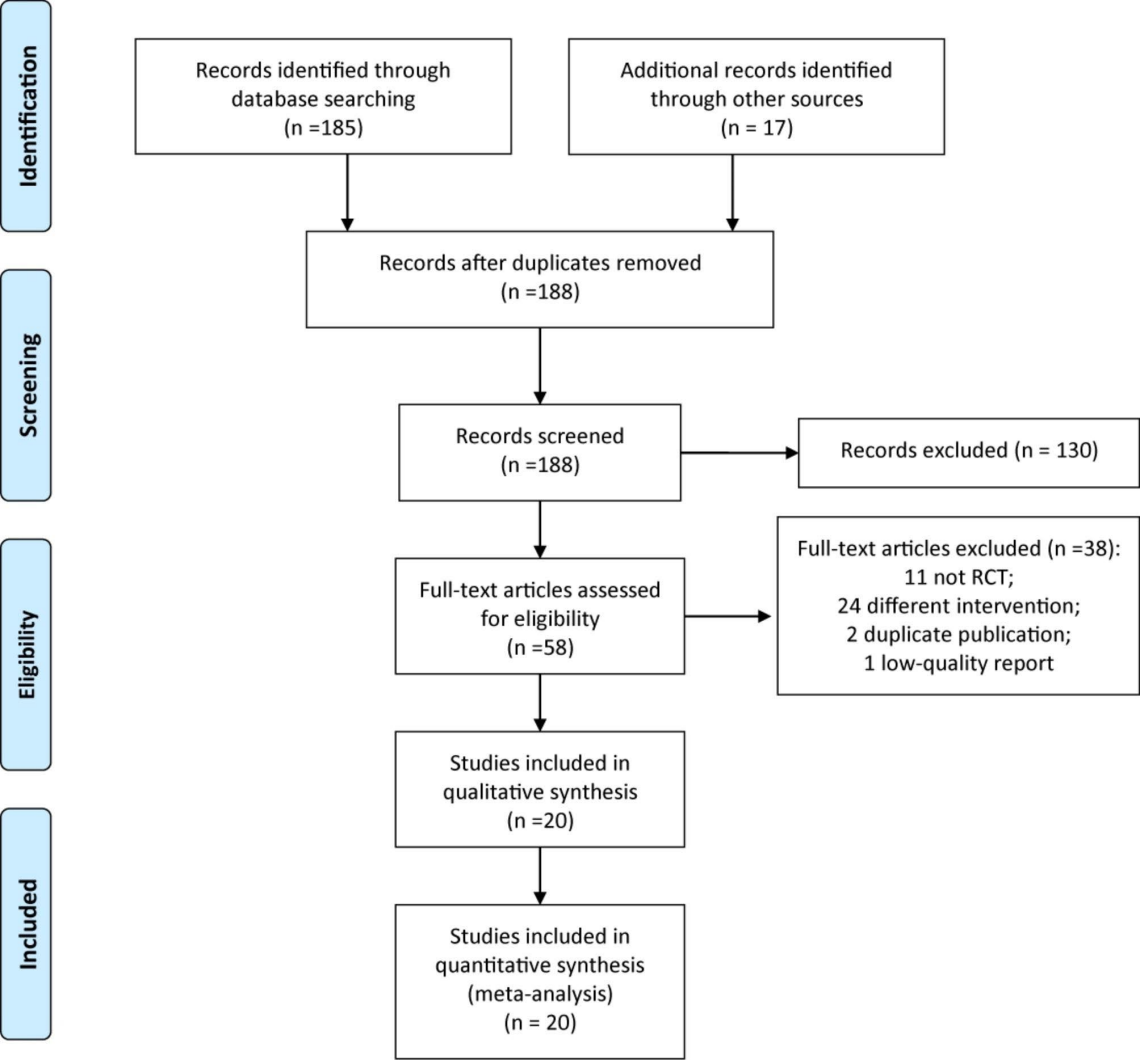
PRISMA flow diagram of RCT selection

A preliminary search obtained 202 relevant literatures. After screening according to the inclusion and exclusion criteria, a total of 20 RCTs [18–37] that met the criteria were finally included in this meta-analysis. The literature screening process is shown in Fig. 1.

### Characteristics of included studies

Of the included 20 RCTs [18–37], a total of 2306 patients with hypertension were included, of whom 1154 patients received music therapy, 1152 received routine anti-hypertension treatment. The characteristics of the included studies are shown in Table 1.

**Table 1:** The characteristics of included studies

Study ID	Country	Sample size			Intervention		Frequency and duration	Outcomes
		Music group	Control group		Music group	Control group		
Bekiroğlu 2013	Turkey	30	30		Music therapy	Rest	25 min per time, once a day, for a total of 28 days	①②⑥
Chen 2009	China	53	53		Music therapy + routine antihypertensive medication and health education	Routine antihypertensive medication and health education	25 m in per time, once a day, for a total of 10 days	①②
Huang 1995	China	20	20		Music therapy	Rest	30 min per time, once a day for a total of 12 days	①②④⑥
Kunikullaya 2015	India	46	47		Music Therapy + lifestyle Intervention	lifestyle Intervention	Each time > 15 min, > 5 times a week, a total of 3 months	①②④⑥
Lei 2013	China	36	36		Music therapy + routine antihypertensive medication	Routine antihypertensive medication	60 min per time, once a day for 12 weeks	①②⑧
Li 2006	China	53	47		Music therapy + routine antihypertensive medication and health education	routine antihypertensive medication and health education	30 min per time, once a day, for a total of 3 months	④
Liang 2018	China	39	39		Music therapy + comprehensive intervention	Comprehensive intervention	30 min per time, once a day, a total of 6 months	①②④⑤⑦
Liu 2014	China	27	27		Music therapy + amlodipine besylate oral therapy	Amlodipine besylate oral therapy	30 min per time, once a day for a total of 30 days	①②③⑩
Lu 2018	China	47	47		Traditional Chinese Medicine Five Elements Music Therapy + routine antihypertensive medication	Routine antihypertensive medication	30 min per time, 2 times a day for 3 months	①②⑥⑦
Shankar 2020	India	100	100		Indian classical music therapy	Routine antihypertensive medication	15 min per time, once a day for a total of 30 days	③
Song 2015	China	60	60		Music therapy + routine antihypertensive medication	Rest + routine antihypertensive medication	30 min per time, once a day for a total of 8 days	①②④⑤⑨
Supap 2018	Thailand	57	57		Thai folk music therapy + routine antihypertensive drug treatment and health education	Routine antihypertensive drug treatment and health education	32 min per time, once a day for a total of 30 days	①②
Tang 2015	China	62	62		Music Therapy + Oral Amlodipine Besylate Tablets and Bisoprolol Fumarate Tablets	Oral Amlodipine Besylate Tablets and Bisoprolol Fumarate Tablets	30 min per time, once a day for a total of 30 days	①②③
Teng 2007	China	12	14		Music therapy	Sit and rest	25 min per time, once a day, for a total of 28 days	①②
Wang 2009	China	50	50		Personalized music therapy + routine basic antihypertensive therapy	Routine basic antihypertensive therapy	50 min per time, once a day for a total of 14 days	①②③④⑤⑩
Yu 2002	China	50	50		Music therapy + routine antihypertensive therapy	Routine antihypertensive therapy	30 min per time, once a day for a total of 30 days	①②④
Zanini 2009	Portugal	23	22		Music therapy + routine antihypertensive medication, regular counseling and health education	Routine antihypertensive medication, regular counseling and health education	60 min per time, once a week for 12 weeks	①②⑧
Zhang 2015	China	49	51		Music therapy + diet and exercise prescription intervention	Diet and exercise prescription intervention	50 min per time, 3 times a day, a total of 28 days	⑧
Zhang 2018	China	100	100		Five Elements Music Therapy + routine drug therapy	Routine drug therapy	30 min per time, 2 times a day, a total of 30 days	①②④⑤⑨
Zhang 2021	China	240	240		Traditional Chinese Medicine Five Elements Music Therapy + routine antihypertensive medication	Routine antihypertensive medication	30–45 min per time, 3 times/week, for at least 8 weeks.	①②④⑤

Notes: ①SBP, ②DBP, ③HR, ④SAS, ⑤SDS, ⑥HAMA, ⑦PSQI, ⑧SF − 36, ⑨Sleep condition, ⑩Symptoms

### The quality of included studies

**Fig. 2:**
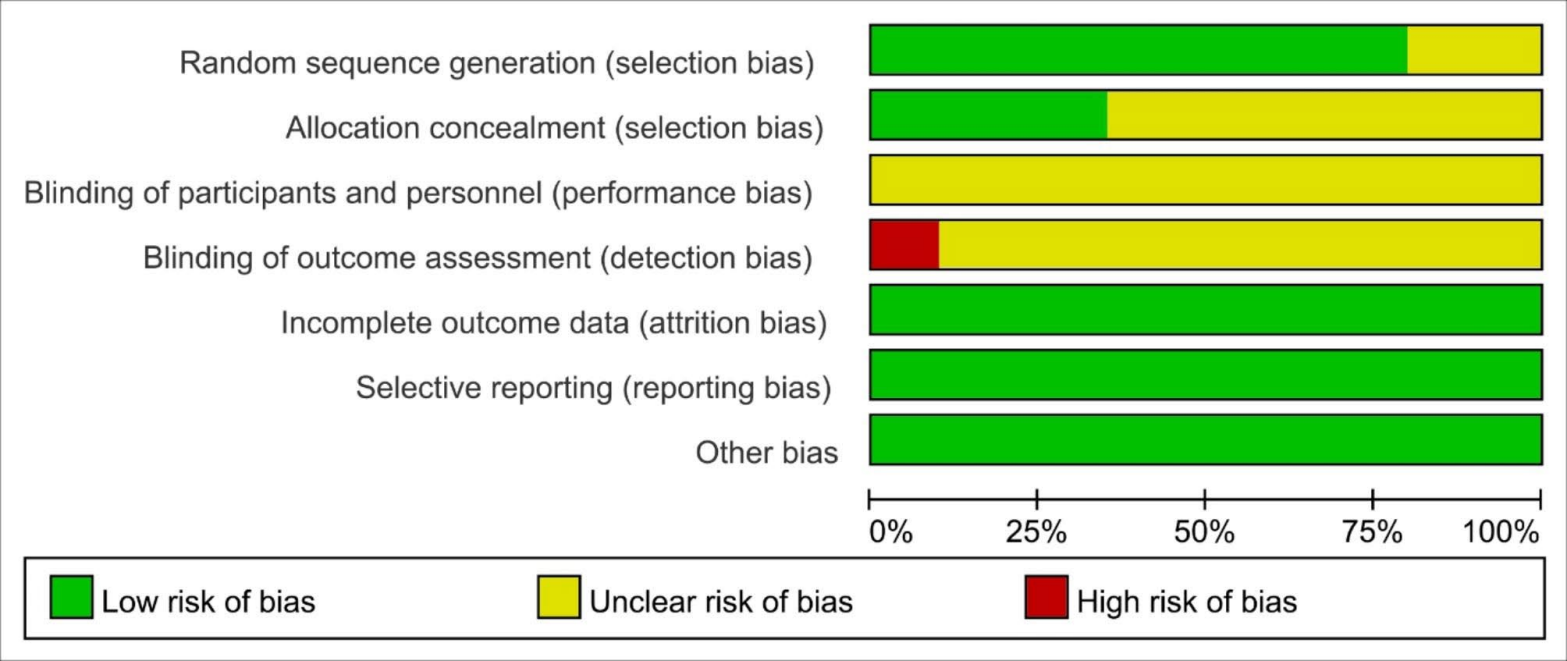
Risk of bias graph

**Fig. 3:**
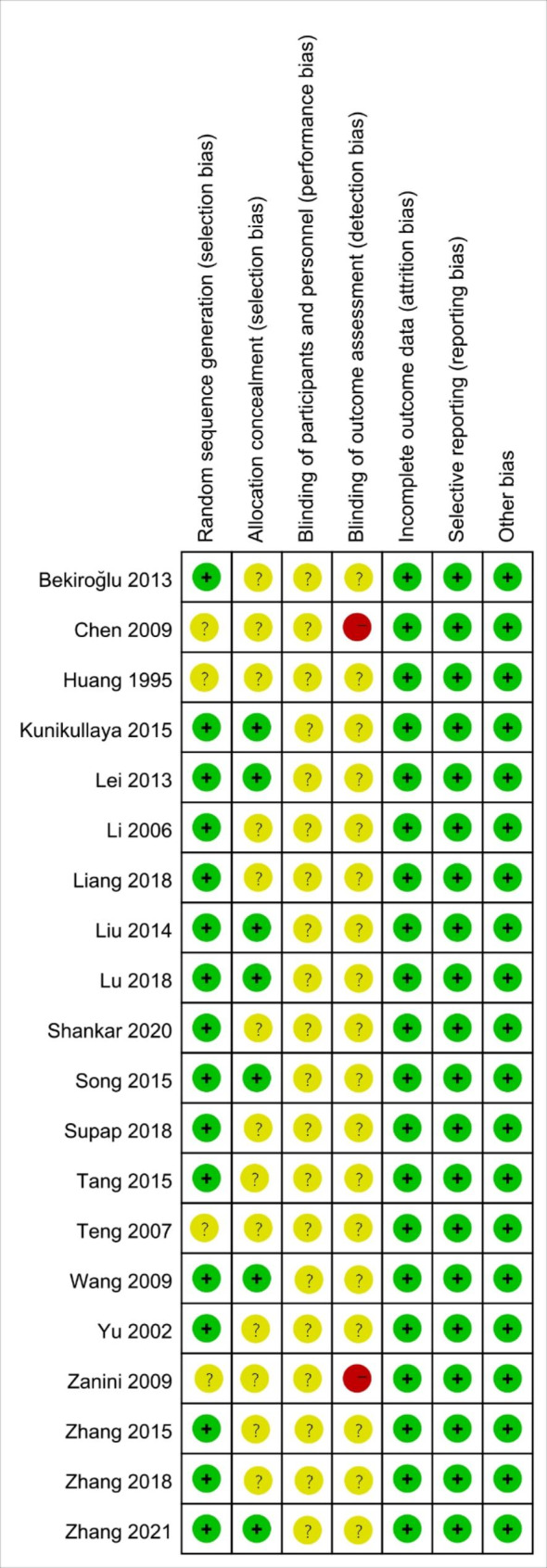
Risk of bias summary

The quality of included RCTs is presented in Figs. 2 and 3. The quality of the studies included in the RCTs was generally good taking into account the integrity of research design and data. Most of the RCTs reported the detailed random sequence generation methods, and random assignment concealment and blinding settings were less reported. Given the nature of music interventions, it was difficult to blind researchers. No bias on other items was found.

### Meta-analysis

#### Systolic blood pressure

**Fig. 4:**
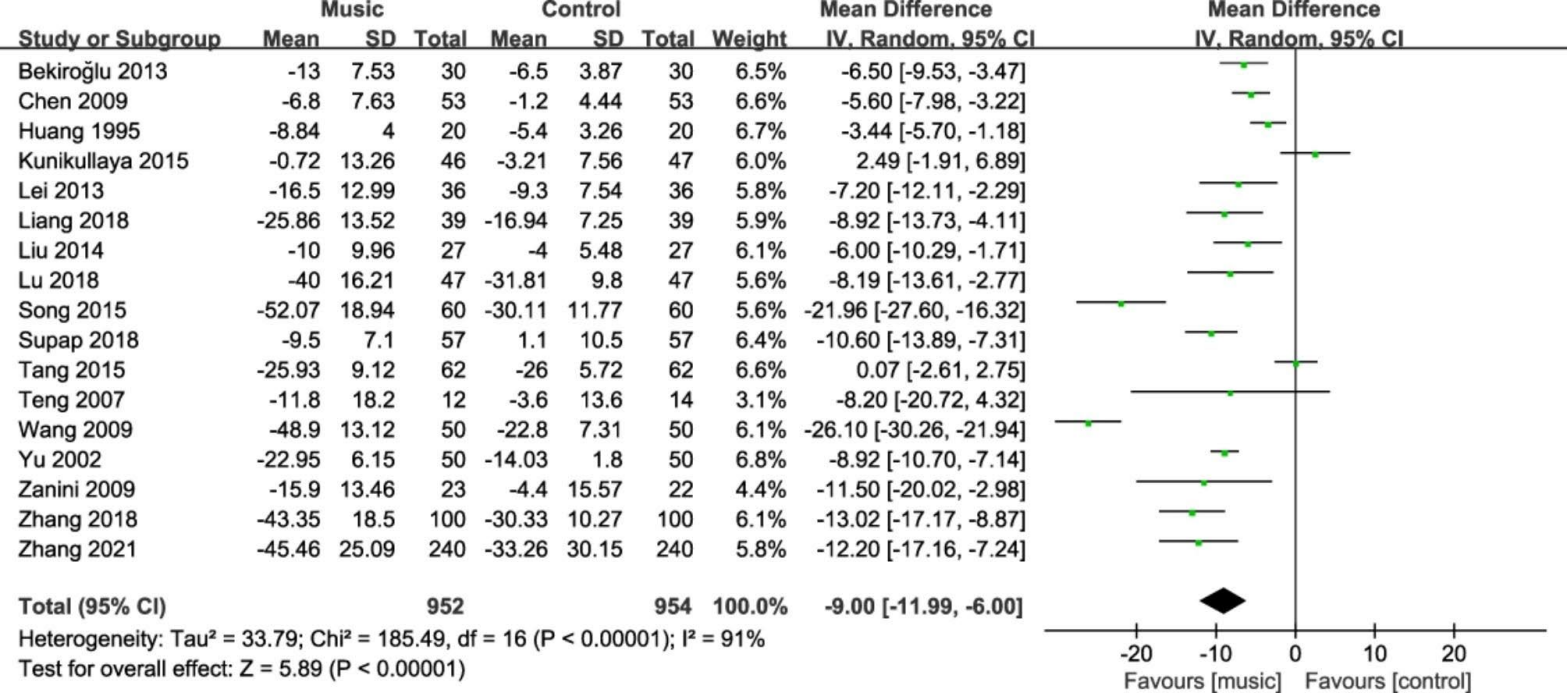
Forest plot for systolic blood pressure change

Seventeen studies [18–20, 22–28, 30–35, 37] reported changes in systolic blood pressure in patients before and after the intervention. The pooled results on systolic blood pressure results were heterogeneous (I^2^ = 91%), so a random-effects model was used to pool the results. The results of meta-analysis showed that the mean systolic blood pressure drop in the music therapy group was greater than that in the control group, and the difference was statistically significant (MD = − 9.00, 95%CI: − 11.99~- 6.00, P<0.001, Fig. 4).

#### Diastolic blood pressure

**Fig. 5:**
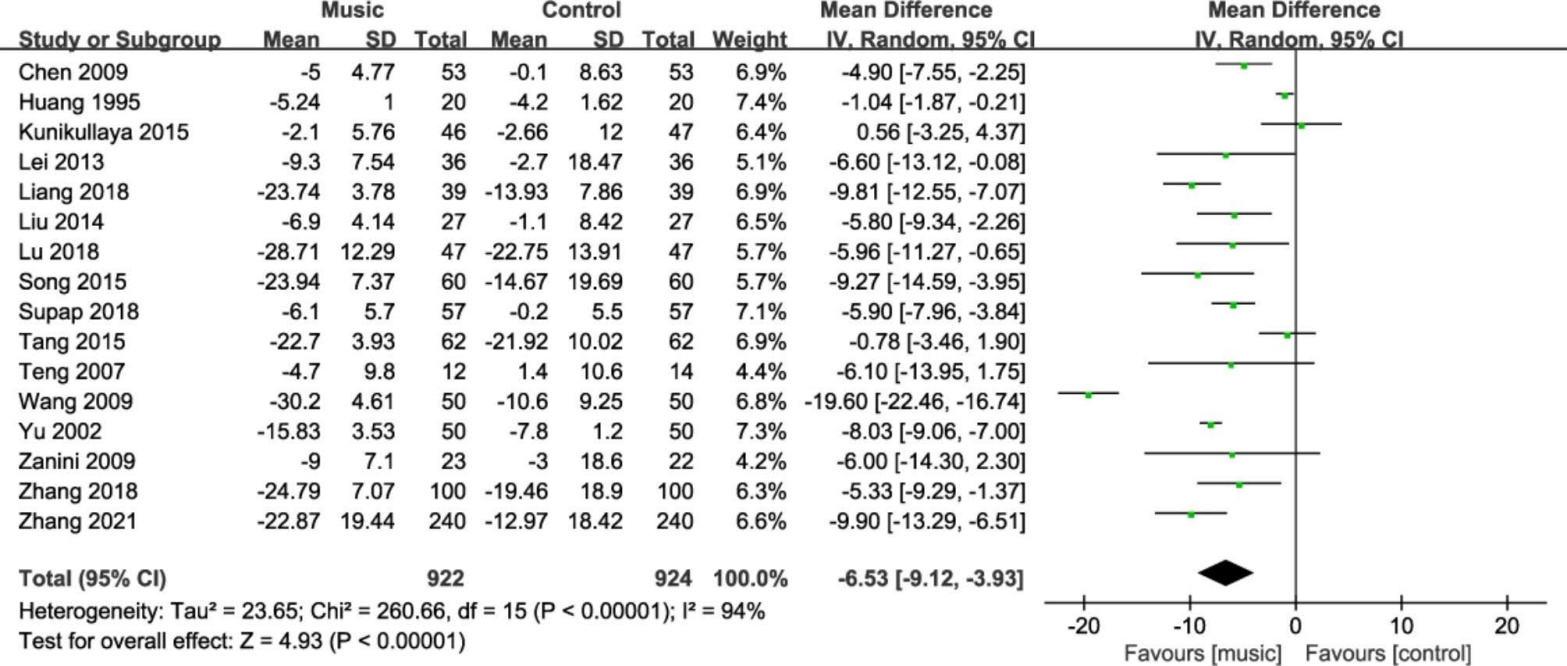
Forest plot for diastolic blood pressure change

Sixteen studies [19, 20, 22–28, 30–35, 37] reported changes in diastolic blood pressure in patients before and after the intervention. The pooled results on diastolic blood pressure results were heterogeneous (I^2^ = 94%), so a random-effects model was used to pool the results. The results of meta-analysis showed that the mean diastolic blood pressure drop in the music therapy group was greater than that in the control group, and the difference was statistically significant (MD = -6.53, 95%CI: -9.12~- 3.93, P<0.001, Fig. 5).

#### Heart rate

**Fig. 6:**
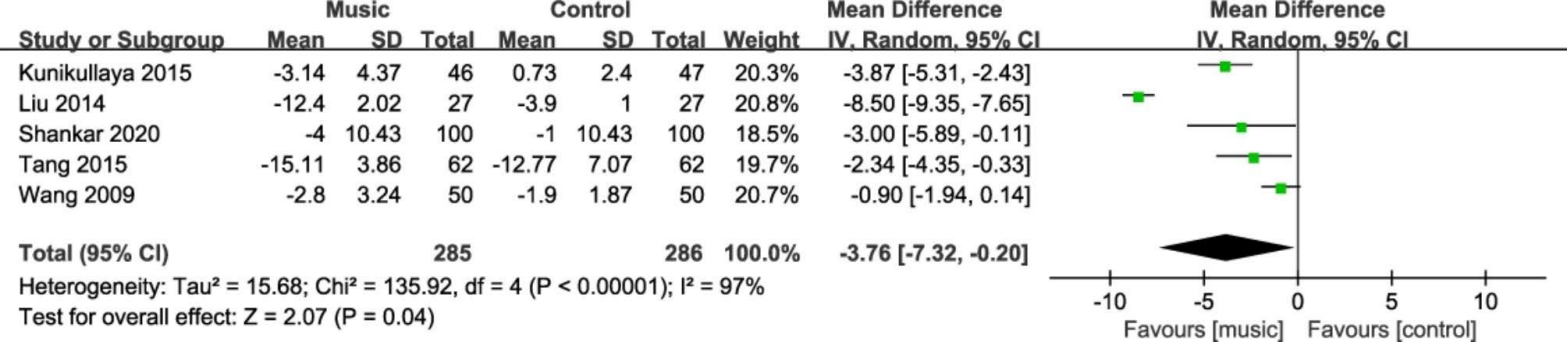
Forest plot for heart rate change

Five studies [24, 25, 29, 33, 37] reported changes in heart rate in patients before and after the intervention. The pooled results on diastolic blood pressure results were heterogeneous (I^2^ = 97%), so a random-effects model was used to pool the results. The results of meta-analysis showed that the mean heart rate drop in the music therapy group was greater than that in the control group, and the difference was statistically significant (MD = -3.76, 95%CI: -7.32~- 0.20, P = 0.04, Fig. 6).

### Other outcomes

As indicated in Table 2, the results of meta-analysis showed that music therapy reduced the SAS score(MD =-8.55, 95%CI: -12.04~-4.12), SDS score(MD = -9.17, 95%CI: -13.85~-5.18), HAMA score(MD = -3.37, 95%CI: − 5.38~- 1.36), PSQI score(MD =-1.61, 95%CI:-2.30~- 0.93) compared with routine therapy in patients with hypertension(all P < 0.05).

**Table 2:** The synthesized outcomes for the effects of music therapy

Outcome	Number of included studies	Heterogeneity	Effect model	MD	95%CI	P
SAS score	7	83.6%	Random	-8.55	-12.04~-4.12	0.04
SDS score	5	90.1%	Random	-9.17	-13.85~-5.18	< 0.01
HAMA score	2	56.4%	Random	-3.37	− 5.38~- 1.36	< 0.01
PSQI score	2	43.3%	Fixed	− 1.61	− 2.30~- 0.93	< 0.01

Notes: SAS, self-rating anxiety scale; SDS, self-rating depression scale; HAMA, Hamilton anxiety scale; PSQI, Pittsburgh sleep quality index

### Publication bias

**Fig. 7:**
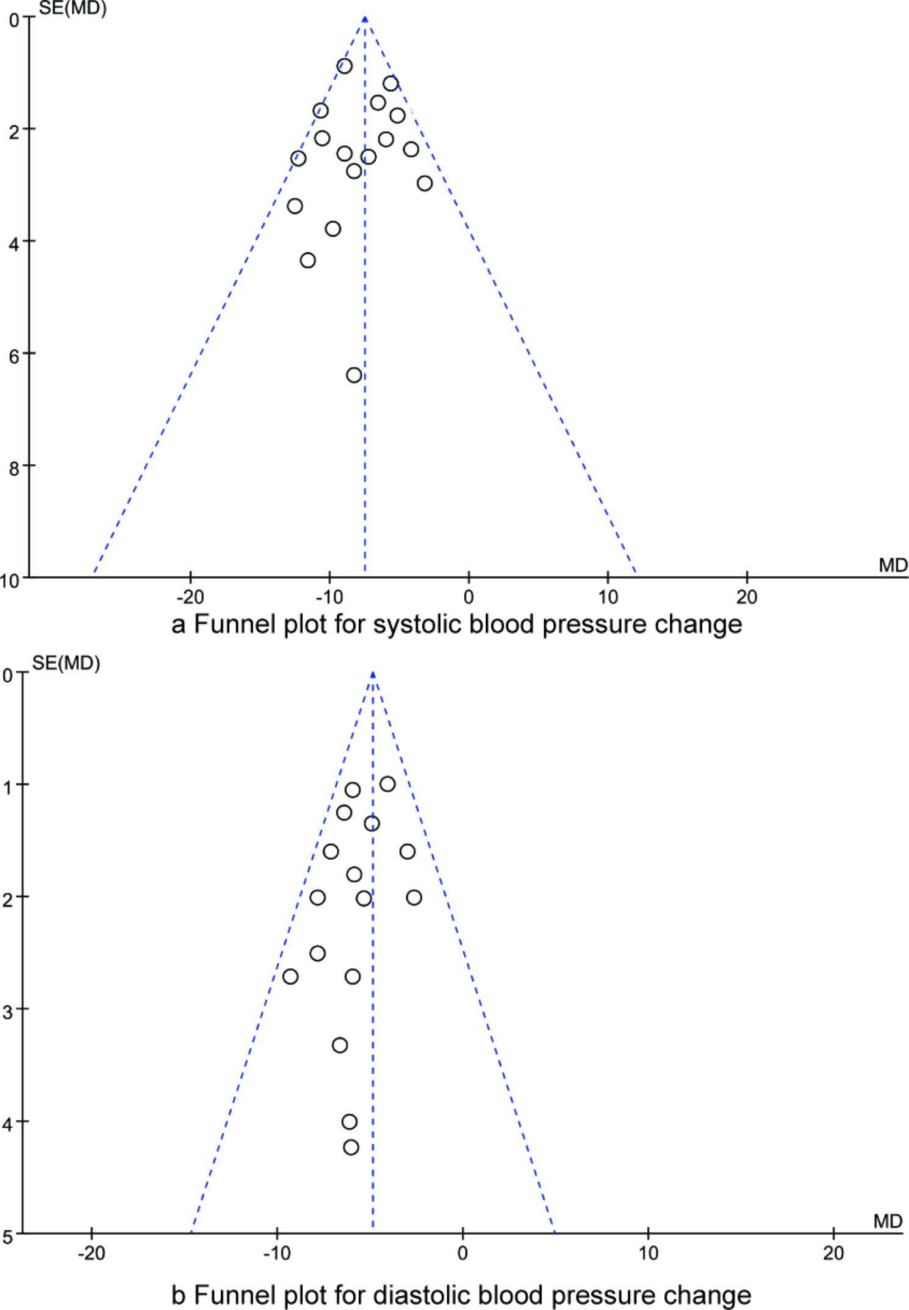
Funnel plots for systolic and diastolic blood pressure changes

We used funnel plots (Fig. 7) combined with Egger’s test to assess the asymmetry of funnel plots, and the results showed that there was no publication bias in each combined result (all P > 0.05).

### Sensitivity analysis

After excluding each study in turn, the heterogeneity and results of the remaining included studies did not change significantly after pooling, indicating that the results were robust and reliable.

## Discussions

Hypertension is a psychosomatic disease related to psychological and mental factors. Long-term mental stress is one of the risk factors for hypertension [38]. The risk of hypertension in people with long-term anxiety and worry is 1.18 times that of normal people [39]. To a certain extent, it is the physiological response of the patient’s nervous and anxious psychological state. A total of 20 RCTs were included in this meta-analysis study, which preliminarily has evaluated the effect of music therapy on blood pressure in hypertensive patients. The results of this meta-analysis indicate that music therapy is beneficial to reduce the systolic, diastolic blood pressure and heart rate, and it is helpful to reduce the anxiety, depression level and improve the sleep quality of patients with hypertension. Music therapy is safe and effective as an adjuvant therapy for hypertension and is worthy of clinical promotion.

The effects and mechanisms of music therapy in the treatment of hypertension may be explained in following aspects. Firstly, it has been reported that when the sound waves of music act on the brain, it increases the excitability of the nervous system and promotes the secretion of hormones such as acetylcholine, thereby slowing down the heart rate [40–42]. Secondly, music diverts the patient’s attention to the disease, reduces sympathetic nerve excitability, produces sedative and antihypertensive effects, and regulates endocrine to reduce renin-angiotensin II secretion to reduce blood pressure [43, 44]. Additionally, the sound wave acts on the brain, adjusts the functional state of the cortex, relieves anxiety and tension, thereby causing changes in physiological and psychological states [45, 46]. In addition, the vitality of the entire nervous system and cell excitability can be enhanced by pleasant music, thereby regulating human physiological activities, eliminating the individual’s tension, reducing their irritable emotions, and ultimately leading to a drop in blood pressure and an improvement in the blood supply function of the heart [47–49].

Music therapy is beneficial to reduce the anxiety, depression level of patients with hypertension. Currently, the pathogenesis of anxiety and depression in hypertensive patients has not been fully elucidated, and it is mostly believed that it is caused by the joint action of serum serotonin (5-HT), norepinephrine (NE) and other neurotransmitters, and is related to neurological and endocrine dysfunction [50]. Both 5-HT and NE are important neurotransmitters in the human body, which can participate in the regulation of various physiological and pathological functions such as body temperature, sleep, mental and emotional functions [51–53]. Studies [54, 55] have found that 5-HT and NE are in low levels in depressed patients. A study [56] has found that compared with before treatment, the levels of 5-HT and NE in the two groups increased after treatment, and the increase in the music therapy group was better than that in the control group. It has been reported that five-element music can improve the level of central neurotransmitter in patients, improve the anxiety and depression state of patients, and then improve the blood pressure control level of patients [57].

Several previous systematic reviews have evaluated the role of music therapy on the patients with hypertension. Yang et al. [58]. have analyzed a total of 7 reports and have concluded that music therapy can effectively reduce diastolic blood pressure in patients, but it does not have effect on and systolic blood pressure. Systolic blood pressure was highly heterogeneous, and no plausible explanation was given for its heterogeneity. Besides, it has been reported that music therapy is only effective for systolic blood pressure, but not significantly for diastolic blood pressure [10]. However, their research only included two literatures for meta-analysis, and it is difficult to support their views due to the small number of sample size, so the conclusions they draw should be treated with caution. Kühlmann et al. [9] have finally included 10 related studies from PubMed, Medline, Cochrane Central, Web of Science and Google Scholar databases, and analyzed that although both systolic and diastolic blood pressure had a downward trend after music therapy, the decline has not reach statistical significance. It has scientific significance and cannot explain the relationship between hypertension and diastolic blood pressure. Compared with published related studies, this study has certain advantages. more RCTs were included than previous studies. Besides, we have chosen stricter inclusion and exclusion criteria, excluding some studies that might affect the accuracy of the results. Furthermore, the evaluation of SAS score, SDS score, HAMA score, PSQI score, etc. of hypertensive patients have been added in this study, and the current research shows that these may be related to the disease control of hypertensive patients.

There are certain limitations in this study that are worth considering. Firstly, most of the included studies lack allocation concealment and double-blind implementation, and there may be selection bias in the time, and type choice of music, which may affect the results of the study. Secondly, some of the included studies did not provide the mean value of the changes before and after intervention, we calculated the coefficient by reporting the complete study, and the results may have a certain bias. Thirdly, some of the included RCTs did not fully report the characteristics of the study population and music type, and the results of some studies were heterogeneous, but there was insufficient data for subgroup analysis, so the results of this study should be treated with caution.

## Conclusions

In conclusion, music therapy can effectively reduce blood pressure and heart rate in patients with hypertension, reduce anxiety and depression levels, and improve sleep quality of patients, thereby improving blood pressure control and prognosis of patients. Compared with drug therapy, music therapy, as a low-cost, easy-to-operate, non-invasive therapy, can reduce medical expenses while achieving therapeutic effects, and it is an adjuvant therapy that clinicians can provide independently. The music therapy is worthy of discussion and application in clinical practice for hypertension management. Still, it is recommended that future studies be designed strictly in accordance with the RCT requirements, and explore the influence of music therapy on more indicators of hypertensive patients, and evaluate the intervention time, music type, playing time, and listening style, so as to provide support for the clinical application of music therapy intervention for hypertensive patients.

## Electronic supplementary material

Below is the link to the electronic supplementary material.

Additional file 1: Supplementary 1. PubMed search strategy table

## Data Availability

All data generated or analyzed during this study are included in this published article.

## Abbreviations

RCTs: Randomized controlled trials
PRISMA: Preferred reporting items for systematic reviews and meta-analyses
CNKI: Chinese National Knowledge Infrastructure
SAS: Self-rating anxiety scale
SDS: Self-rating depression scale
HAMA: Hamilton anxiety scale
PSQI: Pittsburgh sleep quality index
MD: Mean difference
CI: Confidence interval
