# Supplementary material for: Adjuvant music therapy for patients with hypertension: a meta-analysis and systematic review
Source: BMC Complement Med Ther. 2023 Apr 6;23:110. doi: 10.1186/s12906-023-03929-6 (PMC10077636; doi:10.1186/s12906-023-03929-6)
Supplement: Supplementary file 1 — Additional file 1: Supplementary 1. PubMed search strategy table [file 12906_2023_3929_MOESM1_ESM.docx]

Supplementary 1 PubMed search strategy table

| **Database** | **Search strategy** |
| --- | --- |
| Pubmed | #1 " music "[MeSH Terms] OR " sound therapy "[All Fields] OR" music therapy " [MeSH Terms]  #2 " hypertension "[MeSH Terms] OR " high blood pressure "[All Fields] OR " cardiovascular"[All Fields]  #3 #1 or #2  #4 #1 and #2 |
